# The Development of Theory of Mind and Positive and Negative Reciprocity in Preschool Children

**DOI:** 10.3389/fpsyg.2016.00888

**Published:** 2016-06-29

**Authors:** Joanna Schug, Haruto Takagishi, Catalina Benech, Hiroyuki Okada

**Affiliations:** ^1^Department of Psychology, College of William & Mary, WilliamsburgVA, USA; ^2^Brain Science Institute, Tamagawa UniversityTokyo, Japan; ^3^Department of Engineering, Tamagawa UniversityTokyo, Japan

**Keywords:** economic games, reciprocity, theory of mind, preschoolers

## Abstract

This study examined the relation between the acquisition of false-beliefs theory of mind (ToM) and reciprocity in preschoolers. Preschool-aged children completed a task assessing the understanding of false beliefs, and played an Ultimatum Game (UG) with another child in a face-to-face setting. Negative reciprocity was assessed by examining the rejection of unfair offers made by another child in the UG, while positive reciprocity was assessed by examining allocations made by participants in a Dictator Game (DG) following the UG. The results indicated that children who had passed a task assessing first-order false beliefs were more likely to make generous offers in a DG following a fair offer made by their partner in a proceeding UG, but that false beliefs ToM was unrelated to the rejection of unfair offers in the UG.

## Introduction

Reciprocity plays a very important role in the maintenance of human society (Axelrod, 1986; Nowak and Sigmund, 1998; Gintis, 2000; Fehr and Fischbacher, 2003, 2004), and is observed in every human culture (Gouldner, 1960). Individuals can engage in reciprocity either by responding to a positive action with another positive action, or responding to a negative action with a likewise negative action. These two types of reciprocity are known respectively as positive and negative reciprocity. For example, the act of returning a favor would be considered to be positive reciprocity, while punishing criminals for crimes committed would be an example of negative reciprocity.

Overall, research suggests that positive and negative reciprocity (and related concepts such as reward and punishment) may function as independent cognitive mechanisms. A number of studies using behavioral experiments (e.g., Yamagishi et al., 2012), large scale panel data (Egloff et al., 2013), and evolutionary game theory (Szolnoki and Perc, 2013) have suggested that positive and negative reciprocity are distinct and often completely uncorrelated processes. Based on this evidence suggesting that negative and positive reciprocity employ distinct cognitive underpinnings, we sought to examine whether the development of cognitive abilities associated with the understanding of intentions would differentially associate with positive and negative reciprocity in preschool children.

A number of studies have examined factors necessary to create reciprocity, both positive and negative, in interpersonal interactions. One factor which has been found to be very important is intentions (Rabin, 1993; Falk and Fischbacher, 2006), or whether one attributes the cause of an individual’s behavior to their intent of goodwill or malice. People attempt to understand the intentions of others and respond to these intentions in turn by responding altruistically toward other individuals’ benevolent intentions, while punishing and retaliating against hostile intentions. For instance, we take into account intentions of goodwill before we decide to return acts of kindness (McCabe et al., 2003), and in many judicial systems the severity of punishments for different crimes will relate to the intentions of the perpetrator: the unintentional killing of a man will result in a charge of manslaughter, compared with first-degree murder for an intentional killing.

Thus far, experimental studies have provided clear support for intention-based models of reciprocity (Blount, 1995; Falk et al., 2003, 2008; Ohmura and Yamagishi, 2005). One research paradigm that has been extensively used to investigate the importance of intentions on reciprocity, and in particular negative reciprocity, is the Ultimatum Game (UG). The UG is an economic bargaining game widely used to examine the preference for fairness (Güth et al., 1982). The UG is played by two players; the first player, known as the allocator, is given a lump sum of money from the experimenter and is offered the chance to divide this amount between themselves and a second player, known as the responder. The responder then is given the choice of accepting or rejecting the offer. If the responder accepts the proposer’s offer, both players receive the amounts allocated by the first player. However, if the responder rejects the allocation, both parties receive nothing. Thus, rejection in the UG can be thought of as a type of negative reciprocity, in which unfair behavior can be punished.

The ability to make attributions regarding the intentions behind behavior has been found to be extremely important in facilitating reciprocal behavior (Blount, 1995; Falk et al., 2003, 2008; Ohmura and Yamagishi, 2005). Blount (1995) used the UG to investigate whether attributions of intention play a role in negative reciprocity. She compared rejection of unfair offers in an UG in three conditions, a traditional UG in which the first player divides the money between him or herself and the other player, a third part condition in which a neutral third party made the proposal, and a random condition, in which the proposal was purportedly made via a roulette. The results of this study showed that UG responders were significantly more likely to reject unfair offers in the intention treatment than in the non-intention treatment, suggesting that knowing the intentions of others is an important facilitator of negative reciprocity.

The impact of intentions also appears to impact positive reciprocity. Falk et al. (2008) examined the impact of intentions on both positive and negative reciprocity using a simple two-person economic game known as the moonlighting game (MG), which is an extension of the Investment Game (e.g., Berg et al., 1995), with the exception that players can both send and take points from their partner. As in the investment game, points allocated to the other player are tripled. However, players in the MG may also take points from their partner by allocating a negative amount. In this study, participants played a repeated MG either in a treatment where an allocator determined the allocation, or when allocations were determined by a dice roll. The results of this study indicated that intentions were important for both negative and positive reciprocity: players rewarded generous behavior and punished selfish behavior in the intention treatment, but did not engage in any form of reciprocity in the no-intention treatment.

### Theory of Mind and Reciprocity

Understanding the intentions of others requires the ability to infer others’ internal states. This cognitive ability is encapsulated in the notion of *theory of mind* (ToM; Premack and Woodruff, 1978; Baron-Cohen, 2001; Gallagher and Frith, 2003). ToM is a multi-faceted ability that develops across the lifespan beginning in infancy and continuing into adulthood. One facet of ToM that has been studied in the context of economic behavior entails the understanding of false beliefs. False Beliefs Theory of Mind (FB ToM; e.g., Wellman and Liu, 2004), as assessed by passage of tasks which require the understanding of first false beliefs [False Beliefs Task (FBT)], typically emerges in children around 3–4 years of age.^1^

Studies with preschool-aged children have indicated that FB ToM, assessed by passage of a test assessing first-order false beliefs, is strongly related to the propensity to propose fair offers in an UG (Takagishi et al., 2010, 2014)^2^. That is, children who had passed a FBT were more likely to make a fair offer to their partner, while children who have not developed ToM tend to make more selfish offers. These results support the hypothesis that children who are able to successfully pass an assessment of FB ToM are able to understand that another child will reject an unfair offer, and thus make fair offers in the role of the proposer.

Passage of a FB ToM task does not appear to consistently relate to rejection behavior in the UG. Takagishi et al. (2014) showed that responders who were not able to pass a task assessing FB ToM were likely to reject unfair offers, while children who had passed a FB task were more likely to accept unfair offers. Another study (Castelli et al., 2010) examining the relation between FB ToM and rejections of unfair offers in an UG found that children were more likely to reject rather than accept unfair offers even before they were able to pass a false belief task and they were even more likely to reject unfair offers made by a virtual human partner after acquiring FB ToM. Together, these results suggest that the acquisition of FB ToM may not be entirely necessary for the rejection of unfair offers in the UG.

Other studies have examined the role of development in children’s responses to inequitable allocations to the self in economic games. For instance, two studies (Blake and McAuliffe, 2011; Blake et al., 2015) examined the rejection of both advantageous and disadvantageous distributions in children between 4 and 8 years of age found that 4-year-olds were likely to reject unfair offers which were disadvantageous toward themselves. Another study suggests that the moderating effect of intentions on negative reciprocity may develop late in adolescence. Güroǧlu et al. (2009, 2011) conducted studies examining the impact of intentions on rejection in an UG in 10, 13, 15, and 20 year-old participants. They found that younger participants were more likely than older participants to reject unfair offers even when the unfair offers were made unintentionally. Thus, it appears that the importance of intentions in moderating rejection behavior may develop later in life.

In the following study, we sought to examine how the development of ToM, which we operationalize as the understanding of first-order false beliefs, impacts positive and negative reciprocity. The results of previous studies suggest that young children will frequently reject unfair offers, suggesting that the cognitive underpinning related to FB ToM may not be implicated in negative reciprocity, particularly in young children. However, one study examining reciprocity, defined as matching another player’s strategy in a repeated coordination game, found that the tendency for children to match a jointly beneficial allocation increased with age in a sample of children ranging from 3 to 7.5 years of age, an age spanning the typical development of FB ToM (House et al., 2013). Another study found that 5 year-olds, but not 3 year-olds, adjusted their sharing behavior in anticipation of reciprocity (Sebastián-Enesco and Warneken, 2015). However, to the best of our knowledge no study has explicitly examined the relationship between positive and negative reciprocity and the development of FB ToM in children.

A number of studies examining the development of preferences for fairness in children have used the UG (Takagishi et al., 2010, 2014; Blake and McAuliffe, 2011; Blake et al., 2015). However, these studies have found no evidence to suggest that the development of FB ToM plays any role in negative reciprocity among responders in the UG: children who had developed the ability to understand the intentions of others were no more likely to reject unfair offers. In fact, children who had acquired ToM were less likely to reject unfair (unequal) UG offers, an effect Takagishi et al. (2014) suggest may occur due to the children’s desire to maintain harmonious relationships with their peers in non-anonymous games. However, the UG by itself is not well-adapted for examining both positive and negative reciprocity. While the rejection of unfair offers in an UG can be regarded as negative reciprocity or punishment, accepting fair offers cannot be simply regarded as positive reciprocity, as accepting behavior may be motivated purely by self-interest rather than reciprocal motives.

In order to examine the relationship between FB ToM and positive reciprocity, in this study we included a Dictator Game (DG) which took place after the UG. The DG is similar to the UG in that the allocator is given a sum of incentives (in general, money) from the experimenter, and is given the opportunity to split that money between themselves and the recipient in any manner the allocator desires. Unlike the UG, in the DG the recipient has no choice whether or not to accept or reject the offer: they simply receive the amount allocated by the dictator and nothing more. In the current study, the DG took place after the UG, and second players were put into the role of the Dictator to examine their behavior in relation to behavior in the first game. This design allows us to examine positive responses made by the second player in the DG after facing fair and unfair offers in the UG, in addition to rejection behavior in response to fair and unfair offers made by proposers in the DG.

Previous research suggests that behavior in the DG does not consistently relate to development of FB ToM. For instance, Rochat et al. (2009) found that while 5-year-old children made more pro-social allocations in a DG than 3-year-olds, allocations in the DG were not significantly predicted by the ability to understand false beliefs. More recently, Liu et al. (2016) examined the role of the development of various tasks assessing the development of ToM in children aged 3–11, and did not find an association between ToM development and sharing behavior. In contrast, Cowell et al. (2015) found that children who had passed a false beliefs task allocated fewer stickers to an unknown child in another class than did children who did not pass a false beliefs task^3^. Thus, FB ToM may not predict the development of preferences for fairness, but rather the ability to understand that others will reject selfish offers. In other words, the ability for cognitive perspective taking encourages one to behave in a fair manner not because it increases pro-social preferences, but because perspective taking allows one to predict how others will respond to one’s behavior, and behave in manner so as to avoid rejection.

From models of reciprocity which do not distinguish between negative and positive reciprocity (e.g., Rabin, 1993), it can be predicted that the development of FB ToM should increase the amount of rejections of unfair offers in the UG, as well as reciprocity in the subsequent DG. However, because results of previous studies have shown that even young children, who likely have not acquired FB ToM, frequently reject unfair offers in an UG (House et al., 2013) we predicted that FB ToM would impact only positive reciprocity, but not negative reciprocity.

## Materials and Methods

### Ethics Statement

The ethical committee of Tamagawa University approved this study and consent was obtained in advance from parental guardians. The methods were carried out in accordance with the approved guidelines.

### Participants

A total of 108 preschoolers (40 boys and 68 girls) from two preschools in Japan participated in the study in pairs. Children in two sessions (*n* = 4) where one or more of the children did not understand the task or who were not receptive to the experimenter’s guidance were not included in the study. The mean age of the remaining samples was 4.4 years (*SD* = 0.9) and children were from three classes (first year class: 34 girls and 16 boys, mean age = 3.7, *SD* = 0.5, second year class: 28 girls and 18 boys, mean age = 4.7, *SD* = 0.5, third year class: six girls and six boys, mean age = 6.0, *SD* = 0.0).

### Procedures

Preschoolers were recruited for the experiment during free play time by teaching staff, and were led to a private room. All children participated in the experiment in pairs matched by age and gender. To test for acquisition of ToM, we examined childrens’ understanding of first-order false beliefs using an interactive animated version of the false beliefs task (FBT) produced by DIK, Inc.^4^, based on the Sally-Anne task (Baron-Cohen et al., 1985). Prior to beginning of the game, the experimenter individually took the participants to an adjacent room to administer the task on a one-on-one basis using a laptop computer. In this task, participants view a short animation narrated by a computer where a child (named “Natsuki” in the Japanese version) stores a ball in a chest and leaves the room. While Natsuki is out of the room, another child (named “Yuta”) moves the ball to a new location. When Natsuki returns, the program asks children where Natsuki will look for the ball. Participants who have acquired FB ToM should correctly assume that Natsuki will look in the chest where she originally stored her ball. On the other hand, children who have not yet acquired FB ToM will assume that Natsuki will look in the location where Yuta had moved the ball. Prior to commencing the Sally-Anne task, the program instructs children on the vocabulary and objects used in the task (such as the names of the characters, as well as the words “ball” and “box”), and children must correctly identify each object before commencing to the in the task. Participants could respond to the prompt by pointing, responding verbally, or by nodding or shaking their head when the computer offered the options “box” or “bag.”

**FIGURE 1:**
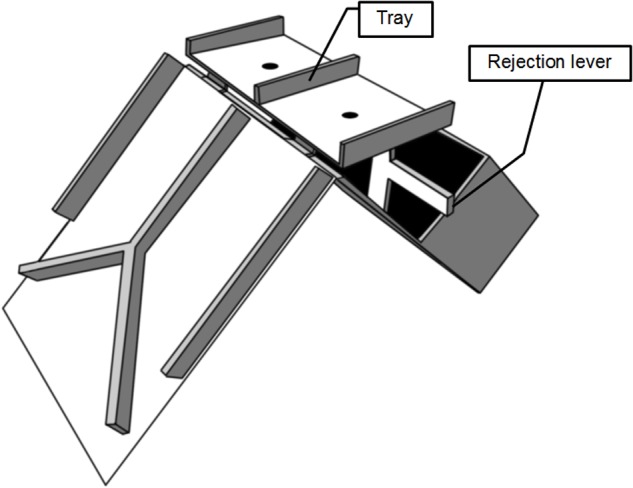
**The apparatus used in the Ultimatum Game (UG).** The proposer sits at the far side and can make an allocation by placing incentives on each side of the divided tray. The responder sits on the near side of the apparatus, and can accept the offer by lifting the tray, or reject the offer using the rejection lever.

After the FBT, participants played an UG using an apparatus (shown in **Figure 1**) which has been used in previous studies (Takagishi et al., 2010, 2014). Children were randomly assigned to the role of the first or second player by asking them to sit, facing each other, on opposing sides of the experimental apparatus. First, the experimenter explained the UG and experimental apparatus (**Figure 1**) to the participants. Colorful fruit-scented erasers, which children highly desired in a pre-experimental pre-test, were used as incentives. After allowing participants to play with the apparatus to understand its functions, the experimenter demonstrated the task by demonstrating all possible divisions of the erasers by placing the fruit-scented erasers on the divided tray of the experimental apparatus. The first player could allocate erasers to herself by placing the erasers on their side of the tray. Alternatively, players could allocate erasers to the second player by placing them on the side closest to the other player.

Next, the experimenter instructed the second player, or responder, on how to accept and reject offers. The second player could accept an offer from the first player by lifting up the tray on which the first player had placed the allocated erasers, in which case the erasers would tumble down a ramp to a container near each participant according to the first player’s offer. Conversely, the second player could choose to reject the offer by pushing down a lever which supported the tray (rejection lever), causing the erasers to fall into a box and be confiscated by the experimenter. The experimenter demonstrated the outcomes associated with all possible combinations of decisions that could be made by the two players in order to ensure that participants understood that they would be able to take home any erasers which fell into their own containers, and that neither child would receive any erasers once they fell into the box. The experimenter repeated the instructions twice and verified children’s understanding by asking them if they understood the task, and continued as necessary until he was satisfied that both children understood the task.

After the instructions, the pair played the UG. The first player was given two erasers by the experimenter, and was asked to make a proposal to the second player regarding how to distribute the erasers by placing them on the tray of the experimental apparatus. The first player thus could make three possible allocations: keep both erasers for him/herself, allocate one eraser each, or give both erasers to the other player. While participants made their decisions, the experimenter turned away from the participants and pretended to be paying close attention to a notebook. By using two erasers as incentives, this design ensured that rejection of unfair offers made by proposers would not entail a cost to the responders. The use of two incentives provides two advantages: first, it allows us to rule out the role of the development of numerical competence on proposals. Second, it allows us to examine only decisions to allocate or not, rather than the decision of how much to allocate, which have been proposed to be two distinct mechanisms (e.g., Blake and Rand, 2010; Liu et al., 2016). After the first player had made their proposal, the experimenter counted the number of erasers allocated to each child out loud, and asked the first player to confirm their allocation. After receiving the confirmation, the experimenter reminded the second player that they could choose to either lift up the tray or push down the lever, and waited for the second player to make their decision. After the second player had made their decisions, the erasers earned by each participant were put into a paper bag with the child’s name written on it.

### Dictator Game

After completion of the UG the participants played a DG. The experimenter removed the experimental apparatus used in the UG and set it aside, leaving only the trays which served as receptacles behind. The experimenter then informed the second player that it was time to play another game. In this game, the second player would decide how to allocate two more erasers between themselves by placing the erasers directly on their own tray and/or the other participant’s tray. The experimenter demonstrated all possible allocations to the second player before handing the child the erasers, and directed his attention away from the children and to a notebook. Again, participants were informed that they would be able to take home any erasers which ended up in their own tray, and the first player would not be able to accept or reject the offer. Participants were not informed of this DG prior to the completion of the UG.

### Relationship Quality

As students engaged in the games in a face to face setting without anonymity, the quality of the relationship between the students playing the games with one another may have an impact on allocation and rejection behavior. Thus, to examine any potential impact of relationship quality on behavior, we asked the children’s home room instructor at their preschool to rate the quality of the relationship between the two children who participated in each game on a scale of 1 (very bad relationship) to 7 (very good relationship).

## Results

### Theory of Mind

Fifty-three of the 104 participants (51%) passed the FBT. The FBT passage rate was positively correlated with age in months (*r* = 0.50, *p* < 0.0001).

### Theory of Mind and Behavior in the Ultimatum Game

First, we examined whether FB ToM related to allocations made by proposers in the UG. Already, several studies (Takagishi et al., 2010, 2014) have found that the development of FB ToM has a positive effect on allocations in the UG. Replicating the results of these studies, participants who had acquired ToM offered more erasers to the second player (β = 0.41, *p* = 0.0028). This result weakened (β = 0.30, *p* = 0.07) when controlling for gender, age in months, and relationship quality. As shown in **Table 1**, allocations were lower among pairs of boys than among pairs of girls^5^ (β = -0.31, *p* = 0.0203). The effects of age in months and relationship quality on allocations made by proposers in the UG were not significant (**Table 1**).

**Table 1:** Relation between assessed variables and UG allocations.

Independent variables	*b*	*SE*	β	*t*	*p*
Age in months	0.01	0.01	0.16	0.65	0.336
Gender (0 = girl, 1 = boy)	-0.34	0.14	-0.31	2.40	0.020
FBT (0 = failed, 1 = passed)	0.32	0.18	0.30	1.84	0.072
Relationship quality	0.02	0.07	0.04	0.34	0.733


Next, we examined whether FB ToM predicted rejection behavior by the second player in the UG. Of the 27 fair and generous offers, 25 were accepted (93%), while 8 of 25 unfair offers (32%) were rejected (Fisher’s exact test; *p* = 0.036). As shown in **Table 2**, FB ToM had a negative, albeit marginal, effect on rejection of unfair offers Wald χ^2^ (df = 1) = 3.44, *p* = 0.06, consistent with the results of a previous study which found that the acquisition of FB ToM corresponded positively with acceptance of unfair UG offers (Takagishi et al., 2014).

**Table 2:** Logistic regression predicting the rejection of unfair offers by responders in the Ultimatum Game.

Independent variables	*b*	*SE*	Wald Chi2	*p*	OR	95%CL
Age in months	0.08	0.07	1.57	0.210	1.09	0.954–1.236
Gender (0 = girl, 1 = boy)	1.21	1.12	1.17	0.280	3.37	0.374–30.33
FBT (0 = failed, 1 = passed)	-2.69	1.45	3.44	0.064	0.07	0.004–1.165
Relationship quality	-1.32	0.68	3.72	0.054	0.27	0.070–1.021


### Theory of Mind and Allocation Behavior in the Dictator Game

Finally, we examined whether the development of FB ToM had any impact on allocation behavior in the DG immediately following the UG. First, we conducted a regression analysis examining the impact of FB ToM, gender, and relationship quality on allocation behavior. The results shown on the left panel of **Table 3**, showed that the acquisition of ToM was positively related to allocation behavior in the DG β = 0.44, *p* = 0.006, and that allocations were lower among pairs of boys than among pairs of girls β = -0.24, *p* = 0.09, while no effects of age in months or relationship quality were observed.

**Table 3:** The impact of ToM and control variables on allocations in the Dictator Game following the Ultimatum Game.

	Step 1			Step 2			Step 3		
			
	*B*	*SE*(B)	β	*B*	*SE*(B)	β	*B*	*SE*(B)	β
FBT (passed = 1)	0.44	(0.15)	0.40^∗∗^	0.29	(0.13)	0.27^∗^	-0.02	(0.14)	-0.02
Age in months	0.01	(0.01)	0.12	0.00	(0.01)	-0.01	0.00	(0.01)	0.02
Gender (boy = 1)	-0.24	(0.14)	-0.22^†^	-0.01	(0.12)	-0.01	-0.05	(0.11)	-0.04
Relationship quality	0.06	(0.07)	0.12	0.03	(0.06)	0.05	0.02	(0.05)	0.04
UG offer				0.65	(0.12)	0.60^∗∗∗^	0.26	(0.16)	0.24
ToM × UG offer							0.61	(0.17)	0.60^∗∗∗^
Intercept	-0.32	(0.46)		-0.06	(0.37)		0.02	(0.33)	
*R*^2^	0.25			0.53			0.63		


*^†^*p* < 0.10, ^∗^*p* < 0.05, ^∗∗^*p* < 0.01, ^∗∗∗^*p* < 0.001.*

However, the purpose of the DG in the current study was not to examine the effect of the understanding of false beliefs on allocation behavior in the DG, but to examine the effect of ToM on reciprocal behavior above and beyond accepting or rejecting offers made by first players in the UG. Because the DG was played immediately following the UG, allocations made are not a pure measure of prosociality, but rather a further measure of reciprocity. Thus, to examine whether the development of ToM had any impact on positive or negative reciprocity in the DG, we included the offer made by the first player in the UG preceding the DG (no erasers offered = 0; 1 or more erasers offered = 1) in the second step of the above regression analysis, controlling for gender, age in months, and relationship quality. The results indicated that the first player’s offer in the UG had a significant impact on the second player’s allocation as a dictator in the DG (β = 0.60, *p* < 0.0001), while the effect of ToM remained significant (β = 0.27, *p* = 0.0235).

**FIGURE 2:**
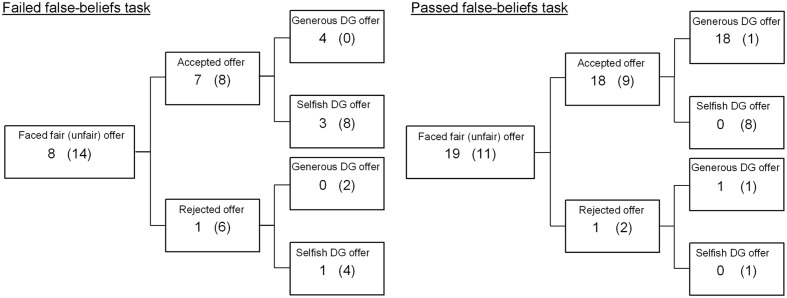
**Counts of participants, by FB ToM passage, who made fair and unfair offers in the Dictrator Game(DG) following the UG.** Counts on the left show number of participants in each category who faced fair offers, and counts on the right in parentheses show counts for participants who faced unfair offers.

Finally, we examined whether the effect of ToM on allocation behavior in the DG was dependent on whether the dictator (second player) had been offered at least one eraser by the first player in the preceding UG. To do so, in the third step we input the ToM × UG offer interaction term in the final step, shown in the third panel of **Table 3**. The results showed that the interaction term was significant (β = 0.60, *p* = 0.0009). Counts of participants who made fair and unfair DG offers after receiving fair and unfair UG offers, by FB ToM passage status, are shown in **Figure 2**. For children who faced a fair or hyperfair offer in the UG, acquisition of FB ToM significantly predicted fair offers in the DG. However, for children who had faced an unfair offer FB ToM did not significantly predict whether or not children allocated an eraser to the other player. Thus, FB ToM predicted DG allocations in cases of positive reciprocity, and was unrelated to negative reciprocity.

## Discussion

The studies presented above examine the impact of ToM, necessary to understand the intentions of others, on positive and negative reciprocity. Consistent with the results of a previous study examining children’s decisions in an UG (Takagishi et al., 2014), the rejection of unfair offers in the UG was not observed more frequently among children who had acquired ToM, as assessed by a FBT. Furthermore, the current study found that children who had acquired ToM were not more likely to respond to unfair offers in an UG with selfish offers in a DG, in which children were given the option to allocate resources between themselves and a partner in any way they pleased. The results of this study, taken together with null and mixed findings related to rejection of unfair offers in the UG observed in previous studies (e.g., Takagishi et al., 2014) suggest that negative reciprocity may be unrelated to the development of FB ToM in preschool children. Regardless of whether they had passed a FBT, children were likely to reject unfair offers and made less equitable allocations in a subsequent DG after being faced with an unfair offer in the UG.

At the same time, this study suggests that ToM plays a role in positive reciprocity: only children who passed the FBT responded to a fair offer in the UG with a fair offer of their own as dictators in the DG. This result suggests that while ToM may not be needed to punish or retaliate against negative behavior, it may increase the tendency for children to respond to being treated in an equitable manner with fair behavior of their own. While the importance of intentions in determining negative reciprocity has been observed in adults, it may be that the ability to inhibit the initial response to reject unfair offers or retaliate against unfair offers may develop later in adolescence (e.g., Güroǧlu et al., 2009, 2011).

This study is not without limitation. One limitation is that the nature of the assessment of FB ToM was dichotomous (i.e., passed/not passed). Future studies should also examine additional measures of cognitive development that are able to account for nuances in the degree to which children have an understanding of false beliefs which may allow for a more detailed interpretation of the impact of FB ToM on positive and negative reciprocity. Studies could seek to examine the development of various components related to ToM, such as second-order FB ToM (e.g., Castelli et al., 2014a,b), perspective taking ability (e.g., Keysar et al., 2003; Fett et al., 2014), and emotional understanding (e.g., Takagishi et al., 2014) on positive and negative reciprocity.

Further research should also attempt to reconcile differences between the results of this and other studies that show that the development of FB ToM in preschoolers is associated with the emerge of strategic behavior in economic games (e.g., Takagishi et al., 2010, 2014), and studies investigating similar tendencies observed in infants and toddlers. Studies of infants show that not only do infants have some understanding of false beliefs and intentions (e.g., Onishi and Baillargeon, 2005; Helming et al., 2014), but that toddlers will also behave in a positive manner toward those who are prosocial, and behave in a negative manner to those who exhibit anti-social behavior (Hamlin et al., 2011). One potential explanation for differences between these studies may involve the willingness of children to give up their personal resources: in this study (as well as many other studies examining prosocial behavior of children in the context of economic games) children must give up their own resources to benefit another, thus future research should examine whether passage of a FBT relates to costly and costless rewarding behavior. In the case of the UG used in this study, rejecting an unfair offer did not incur a cost to participants. In the DG, however, providing one’s partner with an eraser required children to give up a resource that they could have otherwise kept for themselves. Given that prior research has not found consistent evidence linking acquisition of FB ToM with greater allocations in the DG, the finding that DG allocations made after receiving a fair offer in the UG corresponds with the development of FB ToM merits future investigation.

Future studies should further examine the relation between the development of cognitive abilities such as ToM and positive and negative reciprocity using paradigms other than the combination of the UG and DG employed in this study. Indeed, in this study, all participants who accepted unfair offers in the UG allocated both erasers to themselves in the DG, suggesting that even children who accepted unfair offers did not feel that they were treated fairly. The finding may also indicate that children prefer equal outcomes overall. Future studies should be conducted to delineate these and other possibilities. Additionally, this study employed a relatively small sample of participants from a developed country (Japan). Although studies suggest that the development of fairness in young children follows similar trajectories across diverse cultures (e.g., Rochat et al., 2009; Robbins et al., 2015), and that aversion to unfairness toward oneself is found across a number of cultures (Blake et al., 2015), future studies should examine the development of positive and negative reciprocity in larger and more diverse samples.

Overall, this study provides further support for the notion that positive and negative reciprocity are distinct processes (e.g., Yamagishi et al., 2012; Egloff et al., 2013) by showing that the cognitive mechanisms associated with positive reciprocity are not associated with negative reciprocity in preschool-aged children. This approach highlights the utility of examining developmental trajectories in understanding the nature of social decision making in humans (e.g., Gummerum et al., 2008). While the results of this study are preliminary, we hope that future research will examine the role of cognitive abilities and their relation to positive and negative reciprocity over development.

## Author Contributions

JS, HT, and HO, designed and administered the study, JS, HT, and CB conducted data analysis and interpretation, and JS, HT, and CB wrote the paper.

## Conflict of Interest Statement

The authors declare that the research was conducted in the absence of any commercial or financial relationships that could be construed as a potential conflict of interest.

## Footnotes

1 There is considerable controversy in the false beliefs literature regarding the developmental time period associated with the development of the understanding of intentions and false beliefs, and the nature of cognitive abilities measured by FBTs typically used to assess FB ToM in preschool-aged children (see Sabbagh et al., 2013; Helming et al., 2014). While there is a robust finding that preschool aged children become able to pass tests assessing the development of first-order false beliefs in others between 3 and 4 years of age (e.g., Wellman et al., 2001; Wellman and Liu, 2004), other research shows that even infants (e.g., Onishi and Baillargeon, 2005) have some understanding of intentions and can infer the mental states of others. Nevertheless, preschoolers’ passage of FBTs correspond with the development of a number of behaviors, such as lying (e.g., Talwar and Lee, 2008) and secret keeping and playing hide-and-go-seek (Peskin and Ardino, 2003). While this debate is beyond the scope of the current study, we presume that FB ToM reflects the development of cognitive abilities required to act upon the understanding of intentions.

2 Interestingly, second-order false beliefs, which develop later in childhood, do not appear to relate to behavior in the UG (Castelli et al., 2014b).

3 Interestingly, an earlier study (Benenson et al., 2007) had found that DG allocations made to a classmate increased with age among children between 4 and 9 years of age, particularly among high SES children, although this study did not assess ToM. Likewise, studies have shown that found that preschoolers (Fujii et al., 2015) contributed more in the DG played with strangers only when they were being watched by an experimenter (see Takagishi et al., 2015, for similar results with elementary school age boys), or when there is an opportunity for the other player to reciprocate (Xiong et al., 2016). Similarly, Leimgruber et al. (2012) found that 5 years-old were more likely to share only when they knew that the recipient was aware of their donation options. These results support previous work (Castelli et al., 2014b) suggesting that the development of ToM does not relate directly to fairness, but rather increases strategic concerns.

4 http://www.kokoro-cd.com/

5 Note, however, that because pairs were matched by gender, we cannot determine whether gender effects are driven by the gender of the proposer, responder, or a combination of both. Indeed, one recent study demonstrated that gender stereotypes related to the responder influenced proposers allocations in an UG (Fabre et al., 2016), suggesting that the responder’s gender can potentially play a role in influencing the proposer’s allocations.
